# Mental, Neurological, Gastrointestinal, Cardiovascular, and Immunological Impairment After COVID-19 Infection: Case Report and Possible Mechanisms

**DOI:** 10.1192/j.eurpsy.2026.11017

**Published:** 2026-07-31

**Authors:** S. Mihaljevic, D. Weiss-Besic

**Affiliations:** 1 Institute for Public Health Virovitica-Podravina County; 2 Fizioteka, Virovitica, Croatia

## Abstract

**Introduction:**

Survivors of coronavirus disease 2019 (COVID-19) frequently experience a wide spectrum of psychiatric, neurological, gastrointestinal, cardiovascular, and immunological symptoms. These include depression, anxiety, cognitive dysfunction, fatigue, sleep disturbances, gastrointestinal complaints, cardiac arrhythmias, and autoimmune phenomena. While increasing evidence supports the multisystemic impact of SARS-CoV-2, the underlying pathophysiological mechanisms remain only partially understood.

**Objectives:**

In this report, we present the case of a female patient in her mid-40s who developed persistent and multisystemic symptoms—including mental, neurological, gastrointestinal, cardiovascular, and immunological disturbances—following recovery from acute COVID-19. We discuss potential mechanisms underlying these symptoms, drawing on current scientific literature.

**Methods:**

We present a detailed case study of a female patient with a relapsing–remitting post-COVID syndrome. Clinical data were collected through comprehensive medical history, physical and neurological examinations, laboratory and imaging findings, and standardized psychometric assessments. Diagnostic evaluations focused on ruling out alternative etiologies and documenting the multisystem involvement characteristic of long COVID. Follow-up observations were used to track symptom progression and treatment response over time.

**Results:**

Despite comprehensive diagnostic workup, including laboratory and imaging studies, no significant abnormalities were found. The patient reported partial improvement with a multimodal treatment approach that included psychopharmacotherapy (sertraline and low-dose mirtazapine), supportive psychodynamic psychotherapy, functional medicine interventions, mitochondrial support, antihistamine supplementation, and low-dose nicotine therapy.

**Conclusions:**

This case illustrates a chronic, relapsing–remitting form of post-COVID syndrome, marked by substantial impairments in both quality of life and functional capacity. Emerging evidence suggests that SARS-CoV-2 can induce widespread, multi-organ dysfunction via a combination of direct viral injury, persistent inflammation, microvascular damage, immune dysregulation, and disruption of the gut–brain–immune axis. Increasing attention has been given to the roles of neuroinflammation, autonomic nervous system imbalance, alterations in the microbiome, and loss of immune homeostasis in driving the heterogeneous symptom profile of long COVID. Our case provides a compelling example of how these interconnected mechanisms may simultaneously manifest in an individual patient, reinforcing their proposed contribution to the persistence and complexity of long COVID.

**Disclosure of Interest:**

None Declared

